# On the relationship between COVID-19 reported fatalities early in the pandemic and national socio-economic status predating the pandemic

**DOI:** 10.3934/publichealth.2021034

**Published:** 2021-05-24

**Authors:** Kathleen Lois Foster, Alessandro Maria Selvitella

**Affiliations:** 1 Department of Biology, Ball State University, 2111 W. Riverside Ave., Muncie, IN 47306, USA; 2 Department of Mathematical Sciences, Purdue University Fort Wayne, 2101 E. Coliseum Blvd., Fort Wayne, IN 46805, USA

**Keywords:** COVID-19, Socio-economic determinants, spatial effects, cases and deaths, early pandemic, ensemble models, variable selection

## Abstract

This study investigates the relationship between socio-economic determinants pre-dating the pandemic and the reported number of cases, deaths, and the ratio of deaths/cases in 199 countries/regions during the first months of the COVID-19 pandemic. The analysis is performed by means of machine learning methods. It involves a portfolio/ensemble of 32 interpretable models and considers the case in which the outcome variables (number of cases, deaths, and their ratio) are independent and the case in which their dependence is weighted based on geographical proximity. We build two measures of variable importance, the *Absolute Importance Index* (AII) and the *Signed Importance Index* (SII) whose roles are to identify the most contributing socio-economic factors to the variability of the COVID-19 pandemic. Our results suggest that, together with the established influence on cases and deaths of the level of mobility, the specific features of the health care system (smart/poor allocation of resources), the economy of a country (equity/non-equity), and the society (religious/not religious or community-based vs not) might contribute to the number of COVID-19 cases and deaths heterogeneously across countries.

## 1. Introduction

A first episode of pneumonia with unknown cause was detected in Wuhan at the end of 2019 and reported to the World Health Organization (WHO) Country Office in China on 31 December 2019 [1]. This was the first case of the novel coronavirus (SARS-CoV-2) that quickly spread all over the world in the past few months. The WHO declared the outbreak a Public Health Emergency of International Concern on 30th January 2020; on 11th March 2020 WHO further determined that COVID-19 could be characterized as a pandemic [1].

Several studies [2]–[4] have concentrated on the biological and epidemiological factors governing COVID-19 transmission, while few others [5] have investigated the potential impact of socio-economic characteristics on governing the extent of COVID-19 diffusion in the population. Societal and economic factors can be of critical importance for accuracy of models of the outbreak because of the economic [6],[7] and health impacts of the drastic measures that have been put in place in an effort to slow the spread of the disease in those same countries (e.g social distancing, quarantine, lockdowns, testing, and reallocation of hospital resources) [8], [9] and worldwide.

In this paper, we take a reverse perspective and analyze how socio-economic determinants pre-dating the pandemic (data taken not later than 2019) relate to the number of reported cases, deaths, and the ratio detahs/cases of COVID-19 via machine learning methods. Our focus is on understanding the connection between epidemiological variables of the COVID-19 pandemic and the (i) level of health care infrastructure, (ii) general health of the population, (iii) economic factors, (iv) demographic structure, (v) environmental health, (vi) societal characteristics, and (vii) religious characteristics of a country. We hypothesize that different countries have different specific socio-economic features and incidence of the disease and therefore the implementation of government measures must be thoughtful and data evidence-driven and heterogeneous across countries and across resources in order to effectively combat COVID-19.

We analyze 32 interpretable models, including (i) regression models with both independent and proximity dependent outcomes; and (ii) variable selection through LASSO. After this step, we build two indices of variable importance for each of the determinants to estimate their overall association with the the number of cases, deaths, and deaths/cases rate. An *Absolute Importance Index* (AII) and a *Signed Importance Index* (SII) are constructed. The AII determines the presence of the variable in the top-10 absolute correlation ranking, while the SII takes in consideration the sign of the correlation. By focusing only on those findings that are common to a majority, the findings are less sensitive to the limitations of any single model considered.

Our analysis determines that the socio-economic status of a country follows some sort of *Action-Reaction Principle*, as it is not only heavily influenced by the pandemic (we did not address this in the study, but it is an established fact in the literature [10]–[12]), but it is also a distinct factor of the pandemic and must be taken into consideration by governments. The level of mobility, the quality of the health care system, the economic status of a country, and the features of a society are associated with the number of cases and deaths due to COVID-19.

Our work suggests that government resources (both in the form of equipment and staff) must not be allocated blindly, and highlights that different countries might benefit from different measures based on the specific country socio-economic status.

The remaining part of the manuscript is organized as follows. Section 2 is dedicated to a brief description of the datasets used, the description of the epidemiological variables that we are considering, and the description of the socio-economic determinants involved in our study. Moreover, it includes a summary of the methods used. More details on this are present in the Supplementary Material (Appendixes S1, S2, S3, S4). Section 3 is dedicated to the results of our analysis, while Section 4 to the discussion of the results, and Section 5 to the conclusions.

*Remark*. A complete literature review is not feasible, given that the global effort of researchers around the world has produced a massive amount of results on COVID-19. We apologize for all citations that are missing.

## 2. Materials and method

This section is dedicated to (i) the description of the datasets used for the input variables, the outcome variables, and the geographical weighting; and (ii) the methods used for the statistical analysis (linear regression, LASSO, and MICE). We refer to the Supporting Information for more details about the Data Sources (S1), technical results about our statistical methods (S2), and the tables of Descriptive Statistics (S3), and Importance indices (S4).

### 2.1. Datasets

Multiple datasets have been combined for the analysis (See Appendix S1 for the specific information on the data sources, and relative websites). The datasets were then organized in three groups based on their role in the models: (i) the outcome variables **Y**, namely epidemiology variables, such as the number of cases, the number of deaths, and the ratio between them; (ii) the predictors **X** which include the socio-economic determinants; and (iii) the weighting matrix *A*, which includes geographic information.

#### 2.1.1. Outcome variables *Y*: epidemiological variables

The total number of reported cases and deaths attributed to COVID-19 as of 2nd May 2020 were obtained from Our World In Data [13] and used as outcome variables of our models.

#### 2.1.2. Predictors *X*: Socio-economic (SE) factors

As the only predictors of our models, 44 SE variables were chosen for our analyses based on their potential explanatory power and to facilitate comparisons with other published works [5]. Data were obtained from publicly available databases [14]–[20] for a total of 199 countries/regions, 32 of which only had data for all 44 variables of interest (see Appendix S1 for more details). Given the fact that the years for which data were available varied by country/region, we chose to use the most recent data available for each country, (oldest being 2010, most recent being 2019). SE factors were divided into 7 categories based upon the common theme to which each of our 44 variables most closely aligned. These categories are similar to some that have been used previously [5]: (i) Capacity of a country to deal with COVID-19 cases (Healthcare Infrastructure); (ii) Statistics indicative of the health of the population of a country (Health Statistics); (iii) Economic situation and tourism/mobility in a country (Economic Health); (iv) Demographic structure of a country, in particular the age structure and the spatial distribution of the population (Demographic Structure); (v) Societal characteristics such as the level of education, the possibility of access to technology, and features of government (Societal Characteristics); (vi) Pollution level and ecological footprint (Environmental Health); and (vii) Religious practices in the population (Religious Characteristics). The division of variables into each of these categories can be seen in Appendix S1.

#### 2.1.3. Weighting matrix *A*: geographic information

Latitude and longitude coordinates for capital cities were obtained for each country from the CEPII (Centre d'Études Prospectives et d'Informations Internationales) GeoDist database. Coordinates for 11 countries were not found in the GeoDist database and were obtained from Google.

Using these coordinates, we calculated the pairwise distances between cities using the *Spherical Law of Cosines*. Given two cities *C*_1_, *C*_2_ on the surface of the Earth with latitude and longitude coordinates (*α*_1_, *β*_1_) and (*α*_2_, *β*_2_), and assuming a constant radius of the Earth *R* = 6378 kms, we have that the distance between *C*_1_ and *C*_2_ is given by the following formula

$$d(C_{1},C_{2})=arccos(sin(\alpha_{1})*sin(\alpha_{2})+cos(\alpha_{1})*cos(\alpha_{2})*cos(\beta_{2}-\beta_{1}))*R.$$ (1)

The matrix *A* has then been computed such that the entries were given by

$$A_{ij}=\frac{exp\{-d(C_{i},C_{j})\}}{exp\{-d(\cdot ,\cdot )\}}$$ (2)

for *i*, *j* = 1,..., 199. Here *d*(·,·) a normalization factor computed as the average over the distances between every city *C_i_* and every city *C_j_*. We considered the ellipticity of the Earth to have a minor influence on our results and we we considered the spherical approximation appropriate. Our strategy relates to the theoretical work [21].

### 2.2. Methods

#### 2.2.1. MICE

Multiple countries did not report values for some of the SE determinants, so we decided to do imputation, in order to be able to include all countries (both those with and without any missing information). To perform imputation, we used the R package *mice* which performs imputation via *Multivariate Imputation by Chained Equations* (MICE). The method assumes that the probability that a value is missing depends only on observed values and not on unobserved values [22], [23]. We assumed this throughout all our analyses.

The MICE algorithm [22], [23] produces a series of regression models, where each variable with missing data is modeled conditional upon the other variables in the data, and so according to its distribution. In the first step, MICE performs a simple imputation for every missing value in the dataset. The imputations for one of the variables are set back to missing, and this variable is considered as the dependent variable in a regression model with all the other variables used as independent variables, under the assumptions valid in generalized linear models [24]. The missing values for the variable playing the role of dependent variable at this step are then replaced with predictions (imputations) from the regression model. This procedure is then repeated for every variable and in an iterative fashion. At the end of the iteration process, MICE outputs one imputed dataset, and after the process stabilized, the distribution of the parameters governing the imputations is produced. The algorithm is independent on the order in which the variables are imputed. For a summary of the method, we refer to Appendix S2.

### 2.3. Regression models

We considered 5 different outcome variables: (i) *Y*_1_ = # cases; (ii) *Y*_2_ = # deaths; (iii) *Ỹ*_1_ = # cases/total population; (iv) *Ỹ*_2_ = # deaths/total population; and (v) *Y*_0_ = # deaths/# cases. To determine how total population impacted each of the outcome variables *Ỹ*_1_, *Ỹ*_2_, and *Y*_0_ (those dependent variables scaled by population size), we ran each of these models with two different sets of explanatory variables: (i) All variables (|**X**| = 44); and (ii) All but total population (POP) count (|**X**| = 43).

Although the complete dataset contained 199 countries/regions, missing values resulted in preliminary linear regression models excluding 135 of those countries/regions [25]. As mentioned, we imputed the missing values using the *mice* package in R [22] and re-ran our models, so as to include all 199 countries. Models for automatic variable selection, such as LASSO [26], [27] were also considered. We used R Studio Version 1.2.5042 and libraries *readxl*, *readr*, *gdata*, *mice*, *glment*, *caret* for the computations.

### 2.4. Importance indices AII and SII

To measure the importance of the variables across our models, we built two indices. An *Absolute Importance Index* (AII) and a *Signed Importance Index* (SII). The AII counts the number of time a variable appears in the top-10 correlated variables based on absolute correlation. On the other side, the SII counts the presence of a variable in the top-10 correlated variables with sign. For example if the variable *X* appears 12 times in the top-10 highest correlated variables with *Y*, 10 times positively correlated, then *AII* = 12, while *SII* = 8. We computed such indices globally and for each single type of outcome variable. Please refer to Appendix S4 for more details.

## 3. Results

The results of the models produced with the imputed dataset are reported below in Table 1. Multiple and adjusted *R*^2^ values that describe the fit of each model can be found in Table 1 as well. We will describe the specific results about the importance of the variables grouping them by the 7 classes as in Appendix S1, but by joining Health Infrastructures and Health Statistics in one single subsection for convenience of explanation.

**Table 1.** Multiple and adjusted *R*^2^ for all linear regression models performed on the MICE-imputed dataset, containing data for 199 countries/regions. Models that included total population contained 44 socio-economic variables while those excluding total population contained 43. All models were run with and without the weighting matrix *A* based on geographical distance between the largest cities in each country/region. Abbreviations: Pop. Tot. = Population Total; Geo. Weight. = Weighting by Geographical Distance Matrix *A*.

Outcome Variable	With Pop. Tot.	No Geo. Weight.		With Geo. Weight.	
		Mult. *R*^2^	Adj. *R*^2^	Mult. *R*^2^	Adj. *R*^2^
# cases (*Y*_1_)	Y	0.8532	0.8113	0.5626	0.4376
# deaths (*Y*_2_)	Y	0.7918	0.7323	0.6206	0.5122
2*# cases/Pop. Tot. (*Ỹ*_1_)	Y	0.5135	0.3745	0.5225	0.3860
	N	0.5135	0.3785	0.5205	0.3874
2*# deaths/Pop. Tot. (*Ỹ*_2_)	Y	0.4238	0.2592	0.5249	0.3892
	N	0.4235	0.2636	0.5229	0.3905
2*# deaths/# cases (*Y*_0_)	Y	0.2997	0.0996	0.4942	0.3597
	N	0.2973	0.1023	0.4891	0.3474

In all the figures, the results are grouped based on the category. Half of the models included weighting with a geographical distance matrix (see Section 2 for more details). The number of times each variable appeared among the top-10 highest correlated variables in these models was tallied with its own sign (sign + if positively correlated, sign - if negatively correlated). The magnitude and direction of each bar represents the signed percentage of this tally. The letters in the figures represent the way the 44 socio-economic variables are divided into categories. A: Health Infrastructure, B: Health Statistics, C: Environmental Health, D: Economic Health, E: Demographic Structure, F: Societal Characteristics, and G: Religious Characteristics. Tables of detailed raw, signed, and weighted tallies can be found in Appendix S4.

### 3.1. Healthcare infrastructure and statistics

**Figure 1.**
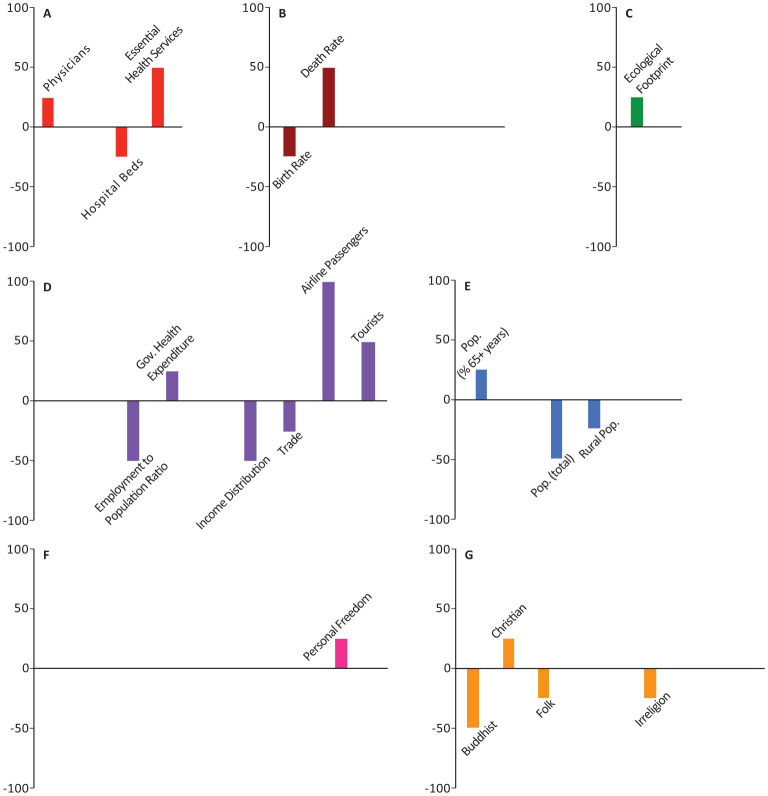
Indices of importance SII of socio-economic variables in models of number of COVID-19 cases (*Y*_1_). We analyzed the effects of 44 socio-economic determinants in a total of 4 models, with number of COVID-19 cases as the outcome variable.

**Figure 2.**
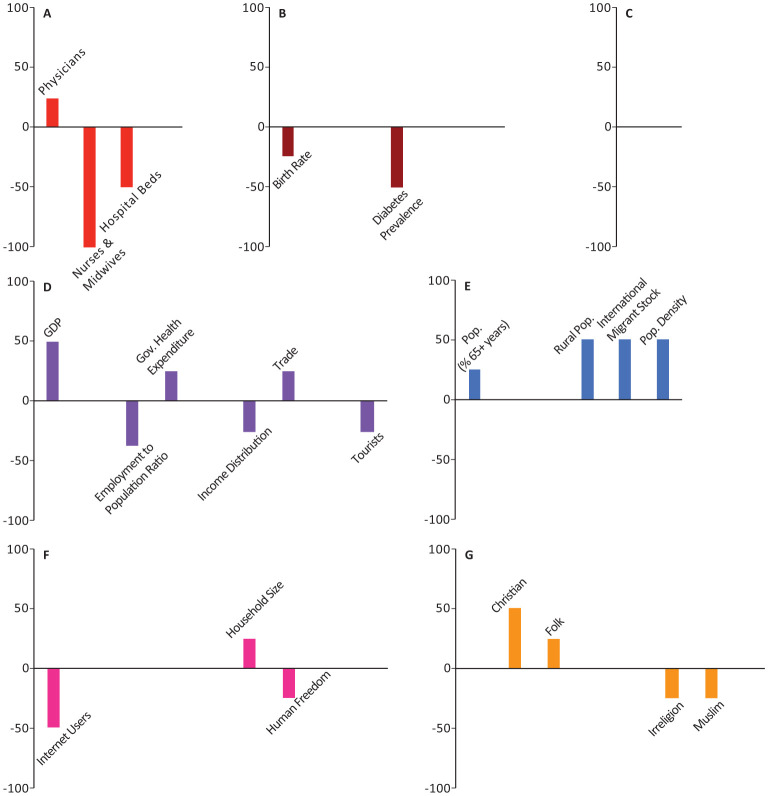
Indices of importance SII of socio-economic variables in models of number of COVID-19 cases/population total (*Ỹ*_1_). We analyzed the effects of 44 socio-economic determinants in a total of 8 models, with number of COVID-19 cases/population as the outcome variable (4 with total population included among the determinants, and 4 with total population removed).

**Figure 3.**
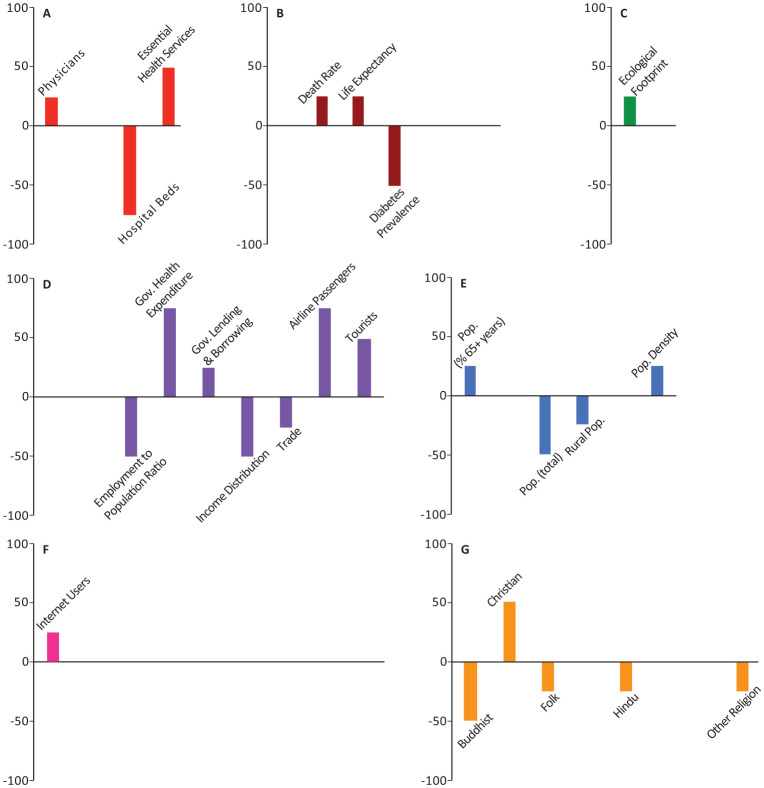
Indices of importance SII of socio-economic variables in models of number of COVID-19 deaths (*Y*_2_). We analyzed the effects of 44 socio-economic determinants in a total of 4 models, with number of COVID-19 deaths as the outcome variable.

**Figure 4.**
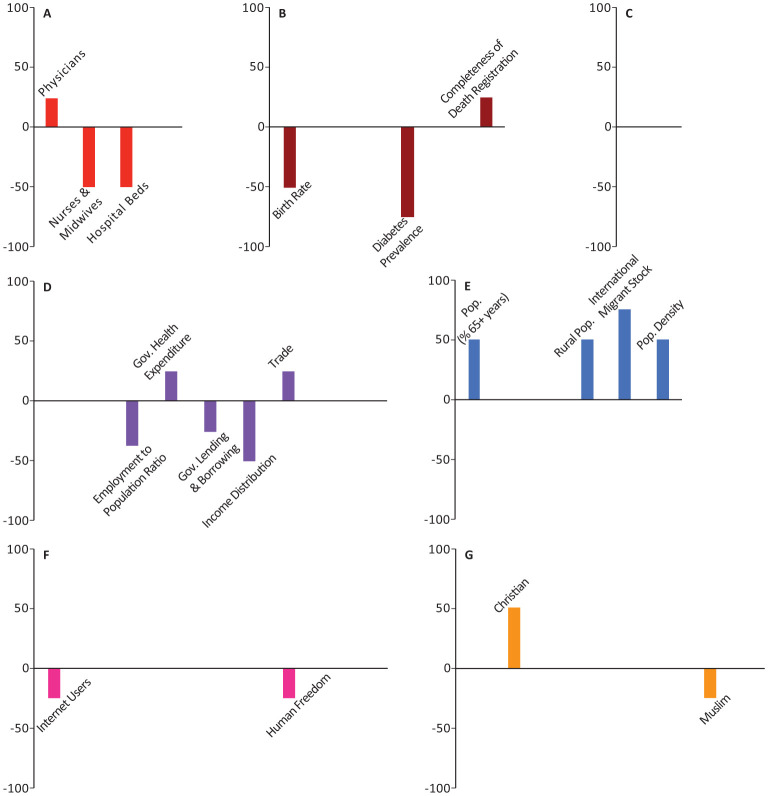
Indices of importance SII of socio-economic variables in models of number of COVID-19 deaths/population total (*Ỹ*_2_). We analyzed the effects of 44 socio-economic determinants in a total of 8 models, with number of COVID-19 deaths/population as the outcome variable (4 with total population included among the determinants, and 4 with total population removed).

**Figure 5.**
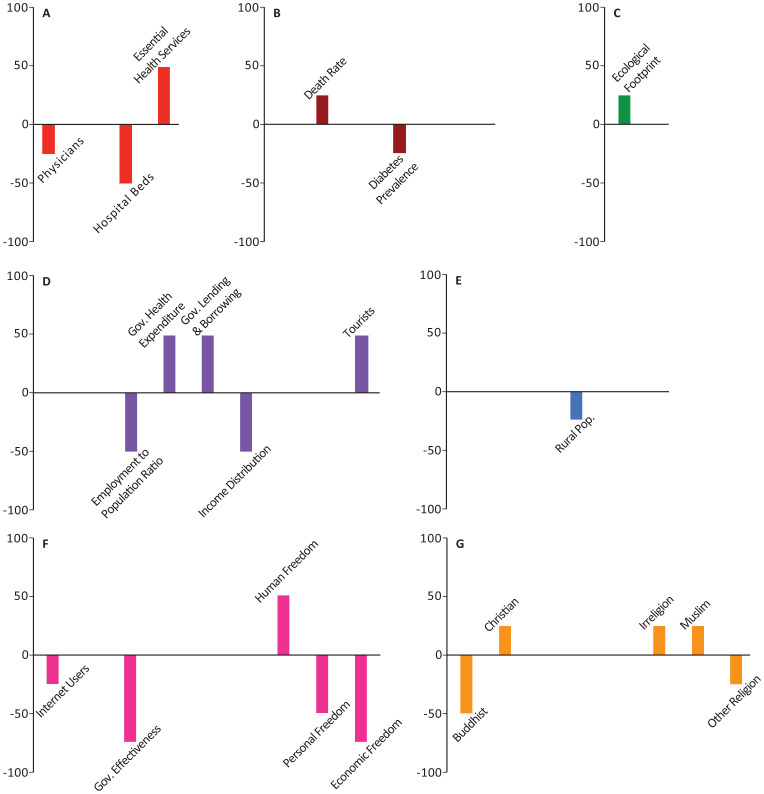
Indices of importance SII of socio-economic variables in models of number of COVID-19 deaths/cases (*Y*_0_). We analyzed the effects of 44 socio-economic determinants in a total of 8 models, with number of COVID-19 deaths/cases as the outcome variable (4 with total population included among the determinants, and 4 with total population removed).

**Figure 6.**
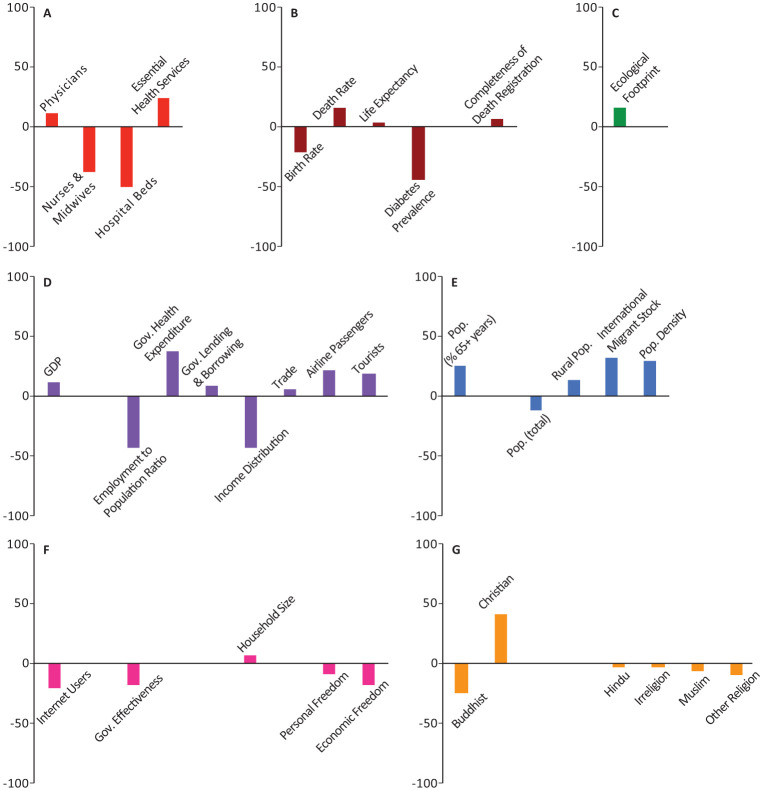
Indices of importance SII of socio-economic variables in all models of COVID-19 cases, deaths, and deaths/cases. We analyzed the effects of 44 socio-economic determinants in a total of 32 models, with number of COVID-19 cases, cases/population total, deaths, deaths/population total, and deaths/cases as outcome variables.

The number of physicians, essential health coverage index, and death rate were among the top-10 variables in 25%, 25%, 15.63% of our models, respectively (Panels A,B in Figures 1–6; Appendix S4). The number of physicians correlated positively with *Y*_1_, *Ỹ*_1_, *Y*_2_, and *Ỹ*_2_ and negatively with *Y*_0_. Access to essential health services consistently correlated positively in our models, appearing in 50% of *Y*_1_, *Y*_2_, and *Y*_0_ models, though completely absent from the top-10 variables in *Ỹ*_1_ and *Ỹ*_2_ models. Similarly, crude death rate correlated positively in 25–50% of *Y*_1_, *Y*_2_, and *Y*_0_ models, but was never selected among the top-10 variables in *Ỹ*_1_ and *Ỹ*_2_ models.

Number of nurses and midwives, number of hospital beds, prevalence of diabetes, and crude birth rate consistently correlated negatively in 37.5%, 50%, 21.88%, and 43.75% of our models, respectively (Panels A,B in Figures 1–6; Appendix S4). Number of hospital beds appeared in all categories of models; it was least common in *Y*_1_ at 25%, most common in *Y*_2_ at 75%, and was in 50% of the models in the remaining categories. Number of nurses and midwives were identified as important in 100% of *Ỹ*_1_ models and 50% of *Ỹ*_2_ models but were notably absent from all other models. Birth rate appeared among our top-10 variables in 25% of *Y*_1_ and *Ỹ*_1_ models and 50% of *Ỹ*_2_ models. Prevalence of diabetes appeared in 25–75% of all model categories, with a notable absence from *Y*_1_ models.

### 3.2. Economic health

Domestic government health expenditure was the only measure of economic health that was identified among the top-10 variables of all categories of models and consistently correlated positively with the outcome variables in all those models (Panel D in Figures 1–6; Appendix S4). In contrast, employment to population ratio and income distribution consistently correlated negatively with the outcome variables in all models and were identified as important determinants in 43.75% of all our models (Panel D in Figures 1–6; Appendix S4). GDP, trade, and government lending/borrowing correlated inconsistently (different for each model) across the models, with GDP and trade only identified as important variables in models lacking the geographical weighting matrix (Panel D in Figures 1–6; Appendix S4). Unemployment rate was never identified as important in any of the models.

### 3.3. Demographic structure and mobility

Total population correlated negatively with *Y*_1_ and *Y*_2_, but became insignificant for *Ỹ*_1_, *Ỹ*_2_, and *Y*_0_ (Panel E in Figures 1–6; Appendix S4). Population density, the proportion of the population over the age of 65, and the proportion of immigrants (people of international stock) consistently correlated positively and were important factors in 28.13%, 25%, and 31.25% of our models, though never in *Y*_0_ (Panel E in Figures 1–6; Appendix S4). Although rural population was identified as an important variable in all categories of models, the pattern of correlation was inconsistent, correlating negatively in *Y*_1_, *Y*_2_, and *Y*_0_ models and positively in *Ỹ*_1_ and *Ỹ*_2_ models (Panel E in Figures 1–6; Appendix S4).

The number of airline passengers per year was nearly always important for *Y*_1_ and *Y*_2_ models, correlating positively in 100% and 75% of those models, respectively, though it never appeared in *Ỹ*_1_, *Ỹ*_2_, or *Y*_0_ models (Panel D in Figures 1–6; Appendix S4). Although the number of tourist arrivals was consistently positively correlated with 50% of *Y*_1_, *Y*_2_, and *Y*_0_ models, the direction of correlation was inconsistent in *Ỹ*_1_ and *Ỹ*_2_, despite being identified as a key determinant in each of those model categories (appearing in 75% and 100% of those models, respectively; Panel D in Figures 1–6; Appendix S4). The geographical weighting matrix was always present in models in which tourist arrivals correlated negatively, while the absence of this matrix in the models always coincided with tourist arrivals correlating positively.

### 3.4. Environmental health

Ecological footprint was identified as an important variable in 25% of *Y*_1_, *Y*_2_, and *Y*_0_ models, in which it consistently correlated positively (Panel C in Figures 1–6; Appendix S4). Air pollution never appeared as an important determinant.

### 3.5. Societal characteristics

The proportion of individuals with access to internet was identified as an important determinant in 28.13% of all our models; in *Ỹ*_1_, *Ỹ*_2_, and *Y*_0_ models, it correlated negatively, whereas in *Y*_2_ models it correlated positively (Panel F in Figures 1–6; Appendix S4). Government effectiveness and economic freedom score correlated negatively with *Y*_0_ and were identified as important in 75% of those models, but no other models (Panel F in Figures 1–6; Appendix S4). Personal freedom also correlated negatively with *Y*_0_ in 50% of those models, though it correlated positively in one *Y*_1_ model (Panel F in Figures 1–6; Appendix S4). Human freedom correlated positively in 50% of *Y*_0_ models, but negatively in 25% of *Ỹ*_1_ and *Ỹ*_2_ models (Panel F in Figures 1–6; Appendix S4). The average number of people per household appeared in 25% of *Ỹ*_1_ models, in which it correlated positively, but was not important in any of the other models (Panel F in Figures 1–6; Appendix S4). Education level, rule of law, and control of corruption never appeared among the top-10 variables in any of our models.

### 3.6. Religious characteristics

The percentage of the population identifying as Christian appeared in 40.63% of models and was a consistently positive correlate in all model categories (Panel G in Figures 1–6; Appendix S4). In contrast, the percentage of the population identifying as Buddhist, appeared in 50% of *Y*_1_, *Y*_2_, and *Y*_0_ models and was a consistently negative correlate in those models (Panel G in Figures 1–6; Appendix S4). The remaining religious categories appeared rarely among the most important variables in our models, but when present, most correlated negatively (Panel G in Figures 1–6; Appendix S4).

## 4. Discussion

We divide our discussion into sections based on the category of socio-economic data. The relationships discussed here are all multivariate correlations, namely correlations occurring in models that contain all socio-economic data.

### 4.1. Healthcare infrastructure and statistics

In countries in which the population has greater access to essential healthcare services and in which the government invests more capital into healthcare, the results surprisingly showed an increase in the number of cases, number of deaths, and number of deaths/cases of COVID-19. In contrast, countries that have greater numbers of nurses and midwives and hospital beds per capita and in which diabetes is more prevalent have smaller numbers of cases, deaths, and deaths/cases. Interestingly, the number of physicians correlated positively with the number of cases and number of deaths, but negatively with the number of deaths/cases. Although seemingly contradictory, taken together, these data may provide indications that government healthcare spending needs to be allocated appropriately in order to effectively combat diseases like COVID-19. This explanation is still not satisfactory, as the possible inefficiencies in the healthcare system do not explain why countries with less capital investment in healthcare would not be struggling with the same inefficiencies. A further reason for this surprising result could be that the population of developed countries is more mobile, and some government epidemic prevention measures are not effective, resulting in higher number of cases and deaths. To narrow down this range of possible interpretations, we would need (among the others) more detailed data about resource allocation to hospitals (e.g. whether or not they experienced shortages of critical equipment). In addition to meeting basic space requirements in the form of hospital beds, access to medical personnel, like nurses and midwives, who interact for greater periods of time directly with patients, may facilitate the treatment of and recovery from both chronic conditions like diabetes and acute conditions like COVID-19. This may be a particularly important consideration for developing countries [6], which may have less effective medical infrastructure in place. The analysis seems also to caution against clustering doctors into large, centralized healthcare facilities when suitable care can be provided at home or in less dense facilities.

### 4.2. Economic health

Employment to population ratio and income distribution, as measured by the GINI index, were identified as important variables in 43.75% of models, and were consistently negatively correlated with number of cases, number of deaths, and number of deaths/cases of COVID-19, both with and without standardization by population. These data suggest that countries in which greater proportions of the population are employed, and where there is less economic disparity within the population, can be expected to feel the effects of COVID-19 less strongly. Indeed, these results appear to be corroborated by the current condition in the United States, a country in which concern about widening economic stratification and discrepancy is frequently discussed and the number of cases and deaths of COVID-19 are continuing to rise rapidly at the time of writing this article (First Trimester of 2021).

Although the roles of GDP and trade were less prominent (appearing in only 12.5% and 6.25% of models, respectively) and were inconsistently correlated with COVID-19 variables, it is important to note that these variables only appeared in models in which there was no weighting by geographical distance. This is interesting given that [5] found that GDP was a strong positive predictor of both COVID-19 cases and deaths. Our results may indicate an influence of geographical clustering of countries with similar economic strength/health, and could potentially be used as an indication of regions in which countries can be expected to exhibit similarities in vulnerability to diseases like COVID-19.

### 4.3. Demographic structure and mobility

Variables relating to demographic structure consistently played a bigger role in models of number of cases and number of deaths than in number of deaths/cases. Indeed, only a single demographic variable (rural population) appeared in only one out of eight of our models in which the number of deaths/cases was the outcome variable. Thus, although the percentage of the population aged 65+, population density, and the percentage of the population of international origin (immigrants) all positively correlated with the number of cases and number of deaths, they do not appear to have an influence in models of the death rate when standardized by the number of cases of COVID-19. Total population correlated negatively with number of COVID-19 cases and deaths (*Y*_1_ and *Y*_2_), but it was no longer identified as an important variable when these outcome variables were standardized by population (*Ỹ*_1_ and *Ỹ*_2_) or in models using deaths/cases (*Y*_0_). This may suggest a sub-exponential growth of the number of infections [30].

The degree of mobility, as indicated by the number of airline passengers, correlated positively with number of cases, number of deaths, and number of deaths/cases, though this effect seemed to disappear in models that were standardized by total population size. Although short-term travel restrictions play an important role in reducing the impact of COVID-19, the fact that the number of airline passengers does not appear in models with outcome variables scaled by population size (*Ỹ*_1_ and *Ỹ*_2_) suggests that increased mobility does not increase COVID-19 cases or deaths to a level disproportionate with the total population of the country. Further, our other variable that directly measures mobility (number of tourist arrivals per year) showed an interesting reversal in correlation structure when weighting by geography was added to our models: tourist arrivals correlated positively in models without the geographical weighting structure, but switched to correlating negatively in all models weighted with geographical data. Interestingly, ecological footprint, which in part could be heavily influenced by domestic mobility (e.g. car travel), appeared in a few models and was consistently positively correlated with cases, deaths, and deaths/cases. Thus, it is clear that the relationship between international travel and COVID-19 is a complicated one, and blanket policies restricting travel may not accurately reflect the impact that such mobility may have on the impact of COVID-19 in national populations.

### 4.4. Religious and societal characteristics

In general, religion, or lack thereof, plays a very minor role in our models, the exceptions being the percentage of the population identifying as Christian, which consistently correlates positively with all our outcome variables, and the percentage of the population identifying as Buddhist, which consistently correlates negatively with all our outcome variables. This is interesting given the speculation early in the pandemic that religious gatherings could be sources of superspreading events [31].

Societal characteristics consistently increase in importance in models in which the number of deaths are standardized by the number of cases compared to models with number of cases or number of deaths as the outcome variables. Further, the majority of the societal variables that correlated strongly in our models had a net negative correlation. In particular, countries in which a greater proportion of their population have greater economic freedom (freedom to voluntarily acquire and dispose of their property [19]), and to a lesser extent personal freedom (freedom of movement, assembly, religion etc. in addition to safety and security [19]), and in which the government is more effective, tend to have lower numbers of deaths/cases. Further, countries in which internet usage is more widespread tend to see a drop in the number of cases, number of deaths, and number of deaths/cases. In contrast, education level, control of corruption, and rule of law never appeared among the top-10 variables in any of our models. Together, these results may suggest that countries with governments capable and effective at enacting policies for the protection of their citizens, who in their turn have the resources to keep themselves informed and freedom to act in their own best interests, may fare better against COVID-19. Note that the implied association between internet access and a more informed public is somehow speculative, possibly non-obvious in the days of misinformation on social media, and certainly requires further attention on its own. It might be more plausible that countries with high levels of internet access are simply in a better position to have a larger number of people work from home, which may not be an option otherwise.

### 4.5. Limitations

Our study is static and photographs the situation at 2nd May 2020. Furthermore, looking at data collected at a single point in time does not take in consideration the fact that the pandemic did not begin to spread in every location simultaneously. For example, European countries began to be affected much earlier than the US. Thus, the strong association between investment into healthcare and number of cases may simply be a consequence of the fact that Europe, a region where healthcare investment is consistently high, was one of the earliest regions to be affected. Also, regions that are affected in the early stages of a pandemic may be hardest-hit even with high capital investment simply because, at that point in time, the medical community still lacks the data and expertise necessary to effectively treat patients. Finally, it may also be that countries with more developed health infrastructure are able to test and diagnose more patients and that COVID-19 deaths will then be more likely to be correctly attributed to the disease.

All of these limitations suggest to follow up this analysis with the analysis of the time evolution of the disease, its relationship with socio-economic covariates, and to account for the possibility of time-lag between different countries.

On another note, it has been shown that underreporting can influence the severity of the pandemic [2], [28], [29], [32]. A future work will incorporate estimates of underreporting in our models.

## 5. Conclusions

In this paper, we have studied the relationship between socio-economic determinants and the reported number of cases, deaths, and the ratio of deaths/cases in each country during the first months of the COVID-19 pandemic, by means of machine learning methods. We analyzed a total of 32 interpretable models and built two importance indices (AII and SII) for the covariates. Our statistical models included linear regression with independent outcomes and geographically weighted outcomes, and variable selection methods such as LASSO. We analyzed the raw data and MICE-imputed datasets.

Our analysis suggests that governments might need to allocate healthcare resources heterogeneously, with a possible benefit in decentralizing healthcare. This could be a problem for developing countries, where the means are limited. As of May 2nd, 2020, countries with more economic equity among their citizens seemed less hit by COVID-19, possibly indicating the importance of having a minimal baseline assistance across the whole population of a country. The analysis of the demographic structure mildly indicated that the disease grows sub-exponentially in the first months of the diffusion. The reduced degree of mobility across countries, for example the degree to which tourism is constrained, had a positive effect in reducing the number of cases, deaths, and death rate per cases. However, there is an indication that a smart and alternating policy could lead to further containment of the disease. Furthermore, our analysis highlighted the benefit of informing the population for government measures to be more effective.

Together, our results seem to indicate that blanket policies are sub-optimal and government measures related to healthcare and immigration have the potential to both help and damage the population, as, if not appropriately taken, they can lead to an increase or reduced decrease of COVID-19 cases, deaths, and deaths/cases rate.
