# Supplementary material for: On the relationship between COVID-19 reported fatalities early in the pandemic and national socio-economic status predating the pandemic
Source: AIMS Public Health. 2021 May 24;8(3):439–55. doi: 10.3934/publichealth.2021034 (PMC8334639; doi:10.3934/publichealth.2021034)
Supplement: Supplementary file 1 [file publichealth-08-03-034-s001.pdf]

Public Health

http://www.aimspress.com/journal/aimsph/

AIMS Public Health, 8(3): 439–455.
DOI:10.3934/publichealth.2021034
Received: 30 March 2021
Accepted: 18 May 2021
Published: 24 May 2021

Research article

On the relationship between COVID-19 reported fatalities early in the pandemic and national socio-economic status predating the pandemic

Kathleen Lois Foster[1] **and Alessandro Maria Selvitella**[2,*]

[1] Department of Biology, Ball State University, 2111 W. Riverside Ave., Muncie, IN 47306, USA
[2] Department of Mathematical Sciences, Purdue University Fort Wayne, 2101 E. Coliseum Blvd., Fort Wayne, IN 46805, USA

* **Correspondence:** Email: aselvite@pfw.edu; Tel: +12604816475.

Supplementary

Appendix S1: Data sources

Supplementary Table of data sources for healthcare, demography, economy and environment		
Variable	*Source*	*Link*
**Healthcare Infrastructure**		
Physicians (/1000 people)	World Bank Open Data	https://data.worldbank.org/
Nurses and midwives (/1000 people)	World Bank Open Data	https://data.worldbank.org/
Hospital beds (/1000 people)	World Bank Open Data	https://data.worldbank.org/
Essential health services (UHC) coverage index	World Bank Open Data	https://data.worldbank.org/
**Health Statistics**		
Birth rate (crude, /1000 people)	World Bank Open Data	https://data.worldbank.org/
Death rate (crude, /1000 people)	World Bank Open Data	https://data.worldbank.org/
Life expectancy at birth (years)	World Bank Open Data	https://data.worldbank.org/
Prevalence of diabetes between ages 20-79 (% population)	World Bank Open Data	https://data.worldbank.org/
Mortality from unsafe water, or sanitation, lack of hygiene combined (/100k people)	World Bank Open Data	https://data.worldbank.org/
Completeness of death registration with cause-of-death information (%)	World Bank Open Data	https://data.worldbank.org/
**Economic Health**		
GDP (per capita, PPP, $)	World Bank Open Data	https://data.worldbank.org/
Unemployment rate (most recent available, % labor force)	International Monetary Fund	https://www.imf.org/en/data
Employment to population ratio for ages 15+ (modeled ILO estimate)	World Bank Open Data	https://data.worldbank.org/
Domestic general government health expenditure (per capita, PPP, $)	World Bank Open Data	https://data.worldbank.org/
Government lending/borrowing (% GDP)	International Monetary Fund	https://www.imf.org/en/data
Income distribution (GINI index)	World Bank Open Data	https://data.worldbank.org/
Trade (% GDP)	World Bank Open Data	https://data.worldbank.org/
Number of airline passengers (per year)	World Bank Open Data	https://data.worldbank.org/
Number of tourist arrivals (per year)	World Bank Open Data	https://data.worldbank.org/
**Demographic Structure**		
Population aged 65+ (% population)	World Bank Open Data	https://data.worldbank.org/
Population aged 0 – 14 (% population)	World Bank Open Data	https://data.worldbank.org/
Population (total)	World Bank Open Data	https://data.worldbank.org/
Rural population (% population)	World Bank Open Data	https://data.worldbank.org/
International migrant stock (% population)	World Bank Open Data	https://data.worldbank.org/
Population density (people per sq km)	World Bank Open Data	https://data.worldbank.org/
**Environmental Health**		
Ecological footprint (gha/person)	Global Footprint Network	http://data.footprintnetwork.org/#/
Air pollution (avg P.M. 2.5 exposure per year)	State of Global Air	https://www.stateofglobalair.org/engage

Supplementary Table of data sources for societal and religious characteristics		
Variable	*Source*	*Link*
**Societal Characteristics**		
Individuals using internet (% population)	World Bank Open Data	https://data.worldbank.org/
Education level: Human capital index (0 – 1)	World Bank Open Data	https://data.worldbank.org/
Government effectiveness	Worldwide Governance Indicators	http://info.worldbank.org/governance/wgi
Rule of law	Worldwide Governance Indicators	http://info.worldbank.org/governance/wgi
Control of corruption	Worldwide Governance Indicators	http://info.worldbank.org/governance/wgi
Avg number of persons per household	United Nations	https://population.un.org/Household/index.html#/countries/840
Human Freedom (score)	CATO Institute	https://www.cato.org/human-freedom-index-new
Personal Freedom (score)	CATO Institute	https://www.cato.org/human-freedom-index-new
Economic Freedom (score)	CATO Institute	https://www.cato.org/human-freedom-index-new
**Religious Characteristics**		
Buddhist (% population)	Pew Research Center - Global Religious Landscape	https://assets.pewresearch.org/wp-content/uploads/sites/11/2014/01/global-religion-full.pdf
Christian (% population)	Pew Research Center - Global Religious Landscape	https://assets.pewresearch.org/wp-content/uploads/sites/11/2014/01/global-religion-full.pdf
Folk Religion (% population)	Pew Research Center - Global Religious Landscape	https://assets.pewresearch.org/wp-content/uploads/sites/11/2014/01/global-religion-full.pdf
Jewish (% population)	Pew Research Center - Global Religious Landscape	https://assets.pewresearch.org/wp-content/uploads/sites/11/2014/01/global-religion-full.pdf
Hindu (% population)	Pew Research Center - Global Religious Landscape	https://assets.pewresearch.org/wp-content/uploads/sites/11/2014/01/global-religion-full.pdf
Irreligion (% population)	Pew Research Center - Global Religious Landscape	https://assets.pewresearch.org/wp-content/uploads/sites/11/2014/01/global-religion-full.pdf
Muslim (% population)	Pew Research Center - Global Religious Landscape	https://assets.pewresearch.org/wp-content/uploads/sites/11/2014/01/global-religion-full.pdf
Other Religion (% population)	Pew Research Center - Global Religious Landscape	https://assets.pewresearch.org/wp-content/uploads/sites/11/2014/01/global-religion-full.pdf

Appendix S2: Statistical analysis

In this file, we collect some technical information in support of the statistical analysis and the results described in the main manuscript of the paper.

Regression methods

In our analysis, we used linear regression methods with independent and dependent outcomes, together with variables selection techniques such as LASSO, complemented with imputation through MICE. Below some details about these methodologies.

Linear regression with independent observations

Consider a data set of n observations $\{\mathbf{x}_i, y_i\} \in \mathbb{R}^p \times \mathbb{R}$. A linear regression model assumes that there is a linear relationship between the outcome variable y and the input variables $\mathbf{x}$, in the form: form

$$y_i = \beta_0 + \beta_1 x_{i1} + \cdots + \beta_p x_{ip} + \varepsilon_i = \mathbf{x}_i^{\mathsf{T}} \boldsymbol{\beta} + \varepsilon_i, \qquad i = 1, \ldots, n,$$

where T denotes the transpose, and $\beta \in \mathbb{R}^p$ is a vector of coefficients. In matrix form, the relationship takes the form

$$\mathbf{y} = X\boldsymbol{\beta} + \boldsymbol{\varepsilon},$$

where

$$\mathbf{y} = \begin{pmatrix} y_1 \\ y_2 \\ \vdots \\ y_n \end{pmatrix} \in \mathbf{R}^n, \quad X = \begin{pmatrix} \mathbf{x}_1^{\mathsf{T}} \\ \mathbf{x}_2^{\mathsf{T}} \\ \vdots \\ \mathbf{x}_n^{\mathsf{T}} \end{pmatrix} = \begin{pmatrix} 1 & x_{11} & \cdots & x_{1p} \\ 1 & x_{21} & \cdots & x_{2p} \\ \vdots & \vdots & \ddots & \vdots \\ 1 & x_{n1} & \cdots & x_{np} \end{pmatrix} \in \mathbb{R}^{n \times p},$$

and

$$\boldsymbol{\beta} = \begin{pmatrix} \beta_0 \\ \beta_1 \\ \beta_2 \\ \vdots \\ \beta_p \end{pmatrix} \in \mathbb{R}^p, \quad \boldsymbol{\varepsilon} = \begin{pmatrix} \varepsilon_1 \\ \varepsilon_2 \\ \vdots \\ \varepsilon_n \end{pmatrix} \in \mathbb{R}^n.$$

The assumptions of the model are the following [22]:

1. **Weak exogeneity**: $\{\mathbf{x}_{1i}, \ldots, \mathbf{x}_{ip}\}$ are nonstochastic variables.
2. **Unbiasedness of the error**: $E[\epsilon_i] = 0$ for every $i = 1, \ldots, n$.
3. **Homoscedasticity**: $Var[\epsilon_i] = \sigma^2$, with $\sigma^2 > 0$ for every $i = 1, \ldots, n$.
4. **Independence of errors** (and outcome variables): $\{\epsilon_i\}_{i=1}^n$ are independent random variables (and so are $\{y_i\}_{i=1}^n$).
5. **Normality**: $\epsilon_i \sim \mathcal{N}(0, \sigma^2)$ for every $i = 1, \ldots, n$.

The parameters of the model can be estimated using ordinary least square methods and produce the explicit formula:

$$\hat{\beta}_{LS} = \left[\mathbf{X}^T\mathbf{X}\right]^{-1} \mathbf{X}^T\mathbf{y} \sim \mathcal{N}(\beta, \sigma^2[\mathbf{X}^T\mathbf{X}]^{-1}),$$

assuming $\mathbf{X}^T\mathbf{X}$ is invertible (namely that that the input variables are not linear combinations of one another) with l_2 error of the order of $n^{1/2}$.

Linear regression with dependent observations

Several of the assumptions of standard linear regression models are too strong, for example the hypothesis of independence between the outcome variables $\mathbf{y}$. It has been shown that if the dependencies are sufficiently weak, then both the coefficient vector β and the strength $\mathbf{A}$ of the dependencies among the response variables can be estimated with an error of the order of $n^{1/2}$, as the Central Limit Theorem guarantees in the case of iid random variables [16].

Our approach including geography dependency is simplified with respect to the framework of [16],

as we assume that **A**, the matrix of geography relationship is known and not to be estimated from the variables $\mathbf{X}, y$. The parameters of the model can be then estimated using again ordinary least square methods and produce a similar explicit formula for the coefficients:

$$\hat{\beta}_{LS} = [\mathbf{X}^T\mathbf{X}]^{-1}\mathbf{X}^T\mathbf{A}\mathbf{y} \sim \mathcal{N}(\beta, \sigma^2[\mathbf{X}^T\mathbf{X}]^{-1}),$$

as

$$E[\hat{\beta}_{LS}] = E[[\mathbf{X}^T\mathbf{X}]^{-1}\mathbf{X}^T\mathbf{A}\mathbf{y}] = [\mathbf{X}^T\mathbf{X}]^{-1}\mathbf{X}^T E[\mathbf{A}\mathbf{y}] = \beta,$$

and

$$\begin{aligned} Var[\hat{\beta}_{LS}] = [\mathbf{X}^T\mathbf{X}]^{-1}\mathbf{X}^T Var[\mathbf{A}\mathbf{y}][[\mathbf{X}^T\mathbf{X}]^{-1}\mathbf{X}^T]^T &= [\mathbf{X}^T\mathbf{X}]^{-1}\mathbf{X}^T\sigma^2\mathbf{I}[[\mathbf{X}^T\mathbf{X}]^{-1}\mathbf{X}^T]^T \\ &= \sigma^2[\mathbf{X}^T\mathbf{X}]^{-1}, \end{aligned}$$

since A is constant with respect to averages and variances taken with respect to the distributions of **X** and **y** by our assumptions.

LASSO

Suppose again to have an sample of n observations, $\{y_i, \mathbf{x}_i\}_{i=1}^n$. Then, the *Least Absolute Shrinkage and Selection Operator* (LASSO) optimizes the following functional [21,22]:

$$J_\lambda(\beta) = \frac{1}{n} \|\mathbf{y} - \mathbf{X}\beta\|_2^2 + \lambda |\beta|,$$

with λ is a pre-specified regularization parameter. The LASSO estimator can be written in explicit form as

$$\hat{\beta}_{LASSO,j} = \begin{cases} y_j - \lambda/2 & \text{if } y_j > \lambda/2 \\ y_j + \lambda/2 & \text{if } y_j < -\lambda/2 \\ 0 & \text{otherwise} \end{cases} \tag{0.1}$$

MICE

For completeness we report here the main details of an algorithm for imputation called *Multiple Imputation by Chained Equations* (MICE), as discussed in [17,18]. Let X_j for $j = 1, \dots, p$ be one of the variables, with X_j^{obs} for $j = 1, \dots, p$ the observed data and X_j^{mis} for $j = 1, \dots, p$ the missing data. Suppose **X** has been partially observed from the multivariate conditional distribution $P(\mathbf{X}|\theta)$ with θ unknown and with its distribution to be determined. MICE samples iteratively through the distributions

$$P(X_j|X_{-j}, \theta_j), \quad j = 1, \dots, p,$$

where X_{-j} is the vector of input variables with X_j dropped. Starting from a simple draw from the marginals, the t-th iteration of the chained equations is a Gibbs sampler that draws

$$\theta_j^{*(t)} \sim P(\theta_j|X_j^{obs}, X_{-j}^{(t-1)}), \quad X_j^{*(t)} \sim P(X_j|X_j^{obs}, X_{-j}^{(t-1)}, \theta_j^{*(t)}),$$

iteratively for $j = 1, \dots, p$. Here $X_j^{(t)} = (X_j^{obs}, X_j^{*(t)})$ is the j-th imputed variable at iteration t. For more details, we refer to [17,18].

Appendix S3: Descriptive statistics

In this appendix, we collect the descriptive statistics of the socio-economic variables and of the epidemiological variables. The values in all the tables have been computed using the raw data (no imputation), which is the reason for the different number of countries per variable.

Descriptive Statistics Table of the Variables divided by Socio-Economic Categories

Variable	*Mean*	*St. Dev.*	*Median*	*IQR*	*Countries*
**Healthcare Infrastructure**					
Physicians (/1000 people)	1.790656	1.584682	1.44805	2.530125	176
Nurses and midwives (/1000 people)	4.21046	4.07985	2.6651	5.362225	176
Hospital beds (/1000 people)	3.053416	2.495021	2.4	2.9	161
Essential health services (UHC) coverage index	64.5954	15.6621	69	24	176
**Health Statistics**					
Birth rate (crude, /1000 people)	19.46667	9.94767	17.0605	16.2905	194
Death rate (crude, /1000 people)	7.616619	2.628278	7.2095	3.39575	194
Life expectancy at birth (years)	72.86528	7.523562	74.3865	10.91167	190
Prevalence of diabetes between ages 20-79 (% population)	7.983505	4.198228	6.8	4.875	194
Mortality from unsafe water, or sanitation, lack of hygiene combined (/100k people)	12.63103	21.13193	1.1	18.325	174
Completeness of death registration with cause-of-death information (%)	88.14912	18.92895	96.5	13	114
**Economic Health**					
GDP (per capita, PPP, $)	22452.38	22512.14	15012.93	26552.91	193
Unemployment rate (most recent available, % labor force)	7.408505	5.072514	6	5.384	107
Employment to population ratio for ages 15+ (modeled ILO estimate)	57.96879	11.5679	58.431	14.835	177
Domestic general government health expenditure (per capita, PPP, $)	982.6385	1293.937	413.1464	1313.689	177
Government lending/borrowing (% GDP)	-2.365106	4.166177	-1.954	4.7405	179
Income distribution (GINI index)	37.9162	7.913178	36.4	10.125	142
Trade (% GDP)	91.39902	52.72362	80.18308	51.38322	182
Number of airline passengers (per year)	27136522	91561319	2118437	13116198	154
Number of tourist arrivals (per year)	7372650	14308081	1783000	6820750	186
**Demographic Structure**					
Population aged 65+ (% population)	8.985213	6.366442	6.954369	11.26835	182
Population aged 0 − 14 (% population)	27.3903	10.47776	25.78098	19.20108	182
Population (total)	37817312	142371892	7650154	25819202	199
Rural population (% population)	40.19289	22.5739	39.681	36.6165	191
International migrant stock (% population)	11.60612	16.49786	4.367492	11.97634	298
Population density (people per sq km)	331.1683	1503.202	90.29942	181.5072	197
**Environmental Health**					
Ecological footprint (gha/person)	3.19826	2.314384	2.447478	2.873057	174
Air pollution (avg P.M. 2.5 exposure per year)	27.79779	19.16504	22.2	23.2	181

Descriptive Statistics Table of Societal and Religious Characteristics					
Variable	*Mean*	*St. Dev.*	*Median*	*IQR*	*Countries*
**Societal Characteristics**					
Individuals using internet (% population)	57.82404	28.63345	64.39999	47.552	195
Education level: Human capital index (0 – 1)	0.5680134	0.1517792	0.576	0.27	149
Government effectiveness	-0.01962567	0.9971855	-0.1	1.245	187
Rule of law	-0.04566845	1.002443	-0.23	1.36	187
Control of corruption	-0.04117647	1.006616	-0.23	1.405	187
Avg number of persons per household	3.941429	1.388127	3.74	2	147
Human Freedom (score)	6.868553	1.096916	6.82	1.515	159
Personal Freedom (score)	6.956161	1.452738	6.933774	2.069753	159
Economic Freedom (score)	6.781069	0.9232247	6.86	1.275	159
**Religious Characteristics**					
Buddhist (% population)	3.80102	14.98954	0	0.225	196
Christian (% population)	58.33531	36.89136	75.6	75.825	196
Folk Religion (% population)	2.303214	6.1582	0.4	1.625	196
Jewish (% population)	0.4749031	5.400602	0	0.00025	196
Hindu (% population)	2.300408	9.809877	0	0.2	196
Irreligion (% population)	8.146888	12.27533	3.2	9.725	196
Muslim (% population)	24.5157	36.41314	3.7	37.75	196
Other Religion (% population)	0.3854082	0.8901687	0.1	0.4	196

Descriptive Statistics Table of COVID-19 reported cases and deaths, and tests at 2nd May 2020					
Variable	*Mean*	*St. Dev.*	*Median*	*IQR*	*Countries*
COVID-19 cases (#)	16612.5	84030.05	690	5389	199
COVID-19 deaths (#)	1197.889	5944.167	16	117	199
COVID-19 deaths/cases	0.04247054	0.04660795	0.03003003	0.05090316	199
COVID-19 tests (#)	337429.1	798274.3	112240	121035.5	24
COVID-19 tests/cases	30.20855	23.0457	25.36336	19.06388	24
COVID-19 tests/deaths	2326.874	3701.545	1005.335	2187.746	24

Appendix S4: Tables of the importance indices

This appendix contains the detailed tables of the importance indices *Absolute Importance Index* (AII) and *Absolute Importance Index* (SII) calculated across all our 32×2 models (geographically weighted + not geographically weighted). Tables with the title "Weighted" refer to the fact that the reported values in those tables are a percentage of the total number of models for that category. For example, there are twice as many models with $\tilde{Y}_1$ as models with Y_1, so transforming the integer scores in percentages corrects for that problem.

AIMS Press

© 2021 the Author(s), licensee AIMS Press. This is an open access article distributed under the terms of the Creative Commons Attribution License (http://creativecommons.org/licenses/by/4.0)

Weighted Index of Importance of Socio-Economic Variables, Divided by Category						
Variable	*Percentage of Models With Variable*					
	Y_1 (4)	$\tilde{Y}_1$ (8)	Y_2 (4)	$\tilde{Y}_2$ (8)	Y_0 (8)	*Total (32)*
**Healthcare Infrastructure**						
Physicians (/1000 people)	25	25	25	25	25	25
Nurses and midwives (/1000 people)	0	100	0	50	0	37.5
Hospital beds (/1000 people)	25	50	75	50	50	50
Essential health services (UHC) coverage index	50	0	50	0	50	25
**Health Statistics**						
Birth rate (crude, /1000 people)	25	25	0	50	0	21.88
Death rate (crude, /1000 people)	50	0	25	0	25	15.63
Life expectancy at birth (years)	0	0	25	0	0	3.13
Prevalence of diabetes between ages 20-79 (% population)	0	50	50	75	25	43.75
Mortality from unsafe water, or sanitation, lack of hygiene combined (/100k people)	0	0	0	0	0	0
Completeness of death registration with cause-of-death information (%)	0	0	0	25	0	6.25
**Economic Health**						
GDP (per capita, PPP, $)	0	50	0	0	0	12.5
Unemployment rate (most recent available, % labor force)	0	0	0	0	0	0
Employment to population ratio for ages 15+ (modeled ILO estimate)	50	37.5	50	37.5	50	43.75
Domestic general government health expenditure (per capita, PPP, $)	25	25	75	25	50	37.5
Government lending/borrowing (% GDP)	0	0	25	25	50	21.88
Income distribution (GINI index)	50	25	50	50	50	43.75
Trade (% GDP)	25	25	25	25	0	18.75
Number of airline passengers (per year)	100	0	75	0	0	21.88
Number of tourist arrivals (per year)	50	75	50	100	50	68.75
**Demographic Structure**						
Population aged 65+ (% population)	25	25	25	50	0	25
Population aged $0-14$ (% population)	0*	0	0	0	0	0*
Population (total)	50	0	50	0	0	12.5
Rural population (% population)	25	50	25	50	25	37.5
International migrant stock (% population)	0	50	0	75	0	31.25
Population density (people per sq km)	0	50	25	50	0	28.13
**Environmental Health**						
Ecological footprint (gha/person)	25	0	25	0	25	12.5
Air pollution (avg P.M. 2.5 exposure per year)	0	0	0	0	50	12.5

*Population aged 0-14 was the first variable identified in LASSO for the geographically weighted $\tilde{Y}_1$, but it dropped out with the addition of the second variable and never returned. Further, it was not identified as important in any other models. Therefore we think it is unlikely that it is a significant variable.

Weighted Index of Importance of Socio-Economic Variables, Divided by Category						
Variable	*Percentage of Models With Variable*					
	Y_1 (4)	$\tilde{Y}_1$ (8)	Y_2 (4)	$\tilde{Y}_2$ (8)	Y_0 (8)	*Total (32)*
**Societal Characteristics**						
Individuals using internet (% population)	0	50	25	25	25	28.13
Education level: Human capital index (0 − 1)	0	0	0	0	0	0
Government effectiveness	0	0	0	0	75	18.75
Rule of law	0	0	0	0	0	0
Control of corruption	0	0	0	0	0	0
Avg number of persons per household	0	25	0	0	0	6.25
Human Freedom (score)	0	25	0	25	50	25
Personal Freedom (score)	25	0	0	0	50	15.63
Economic Freedom (score)	0	0	0	0	75	18.75
**Religious Characteristics**						
Buddhist (% population)	50	0	50	0	50	25
Christian (% population)	25	50	50	50	25	40.63
Folk Religion (% population)	25	25	25	0	0	12.5
Jewish (% population)	0	0	0	0	0	0
Hindu (% population)	50	0	25	0	0	9.38
Irreligion (% population)	25	25	0	0	25	15.63
Muslim (% population)	0	25	0	25	25	18.75
Other Religion (% population)	0	0	25	0	25	9.38

Weighted Signed Index of Importance of Socio-Economic Variables, Divided by Category						
Variable	*Percentage of Models With Variable*					
	Y_1 (4)	$\tilde{Y}_1$ (8)	Y_2 (4)	$\tilde{Y}_2$ (8)	Y_0 (8)	*Total (32)*
**Healthcare Infrastructure**						
Physicians (/1000 people)	25	25	25	25	-25	12.5
Nurses and midwives (/1000 people)	0	-100	0	-50	0	-37.5
Hospital beds (/1000 people)	-25	-50	-75	-50	-50	-50
Essential health services (UHC) coverage index	50	0	50	0	50	25
**Health Statistics**						
Birth rate (crude, /1000 people)	-25	-25	0	-50	0	-21.88
Death rate (crude, /1000 people)	50	0	25	0	25	15.63
Life expectancy at birth (years)	0	0	25	0	0	3.13
Prevalence of diabetes between ages 20-79 (% population)	0	-50	-50	-75	-25	-43.75
Mortality from unsafe water, or sanitation, lack of hygiene combined (/100k people)	0	0	0	0	0	0
Completeness of death registration with cause-of-death information (%)	0	0	0	25	0	6.25
**Economic Health**						
GDP (per capita, PPP, $)	0	50	0	0	0	12.5
Unemployment rate (most recent available, % labor force)	0	0	0	0	0	0
Employment to population ratio for ages 15+ (modeled ILO estimate)	-50	-37.5	-50	-37.5	-50	-43.75
Domestic general government health expenditure (per capita, PPP, $)	25	25	75	25	50	37.5
Government lending/borrowing (% GDP)	0	0	25	-25	50	9.38
Income distribution (GINI index)	-50	-25	-50	-50	-50	-43.75
Trade (% GDP)	-25	25	-25	25	0	6.25
Number of airline passengers (per year)	100	0	75	0	0	21.88
Number of tourist arrivals (per year)	50	-25	50	0	50	18.75
**Demographic Structure**						
Population aged 65+ (% population)	25	25	25	50	0	25
Population aged $0-14$ (% population)	0*	0	0	0	0	0*
Population (total)	-50	0	-50	0	0	-12.5
Rural population (% population)	-25	50	-25	50	-25	12.5
International migrant stock (% population)	0	50	0	75	0	31.25
Population density (people per sq km)	0	50	25	50	0	28.13
**Environmental Health**						
Ecological footprint (gha/person)	25	0	25	0	25	12.5
Air pollution (avg P.M. 2.5 exposure per year)	0	0	0	0	0	0

*Population aged 0-14 was the first variable identified in LASSO for the geographically weighted $\tilde{Y}_1$, but it dropped out with the addition of the second variable and never returned. Further, it was not identified as important in any other models. Therefore we think it is unlikely that it is a significant variable.

Weighted Signed Index of Importance of Socio-Economic Variables, Divided by Category						
Variable	*Percentage of Models With Variable*					
	Y_1 (4)	$\tilde{Y}_1$ (8)	Y_2 (4)	$\tilde{Y}_2$ (8)	Y_0 (8)	*Total (32)*
**Societal Characteristics**						
Individuals using internet (% population)	0	-50	25	-25	-25	-21.88
Education level: Human capital index (0 – 1)	0	0	0	0	0	0
Government effectiveness	0	0	0	0	-75	-18.75
Rule of law	0	0	0	0	0	0
Control of corruption	0	0	0	0	0	0
Avg number of persons per household	0	25	0	0	0	6.25
Human Freedom (score)	0	-25	0	-25	50	0
Personal Freedom (score)	25	0	0	0	-50	-9.38
Economic Freedom (score)	0	0	0	0	-75	-18.75
**Religious Characteristics**						
Buddhist (% population)	-50	0	-50	0	-50	-25
Christian (% population)	25	50	50	50	25	40.63
Folk Religion (% population)	-25	25	-25	0	0	0
Jewish (% population)	0	0	0	0	0	0
Hindu (% population)	0	0	-25	0	0	-3.13
Irreligion (% population)	-25	-25	0	0	25	-3.13
Muslim (% population)	0	-25	0	-25	25	-6.25
Other Religion (% population)	0	0	-25	0	-25	-9.38

Index of Importance of Socio-Economic Variables, Divided by Category						
Variable	*Number of Models With Variable*					
	Y_1 (4)	$\tilde{Y}_1$ (8)	Y_2 (4)	$\tilde{Y}_2$ (8)	Y_0 (8)	*Total (32)*
**Healthcare Infrastructure**						
Physicians (/1000 people)	1	2	1	2	2	8
Nurses and midwives (/1000 people)	0	8	0	4	0	12
Hospital beds (/1000 people)	1	4	3	4	4	16
Essential health services (UHC) coverage index	2	0	2	0	4	8
**Health Statistics**						
Birth rate (crude, /1000 people)	1	2	0	4	0	7
Death rate (crude, /1000 people)	2	0	1	0	2	5
Life expectancy at birth (years)	0	0	1	0	0	1
Prevalence of diabetes between ages 20-79 (% population)	0	4	2	6	2	14
Mortality from unsafe water, or sanitation, lack of hygiene combined (/100k people)	0	0	0	0	0	0
Completeness of death registration with cause-of-death information (%)	0	0	0	2	0	2
**Economic Health**						
GDP (per capita, PPP, $)	0	4	0	0	0	4
Unemployment rate (most recent available, % labor force)	0	0	0	0	0	0
Employment to population ratio for ages 15+ (modeled ILO estimate)	2	3	2	3	4	14
Domestic general government health expenditure (per capita, PPP, $)	1	2	3	2	4	12
Government lending/borrowing (% GDP)	0	0	1	2	4	7
Income distribution (GINI index)	2	2	2	4	4	14
Trade (% GDP)	1	2	1	2	0	6
Number of airline passengers (per year)	4	0	3	0	0	7
Number of tourist arrivals (per year)	2	6	2	8	4	22
**Demographic Structure**						
Population aged 65+ (% population)	1	2	1	4	0	8
Population aged $0-14$ (% population)	0*	0	0	0	0	0*
Population (total)	2	0	2	0	0	4
Rural population (% population)	1	4	1	4	2	12
International migrant stock (% population)	0	4	0	6	0	10
Population density (people per sq km)	0	4	1	4	0	9
**Environmental Health**						
Ecological footprint (gha/person)	1	0	1	0	2	4
Air pollution (avg P.M. 2.5 exposure per year)	0	0	0	0	4	4

*Population aged 0-14 was the first variable identified in LASSO for the geographically weighted $\tilde{Y}_1$, but it dropped out with the addition of the second variable and never returned. Further, it was not identified as important in any other models. Therefore we think it is unlikely that it is a significant variable.

Index of Importance of Socio-Economic Variables, Divided by Category						
Variable	*Number of Models With Variable*					
	Y_1 (4)	$\tilde{Y}_1$ (8)	Y_2 (4)	$\tilde{Y}_2$ (8)	Y_0 (8)	*Total (32)*
**Societal Characteristics**						
Individuals using internet (% population)	0	4	1	2	2	9
Education level: Human capital index (0 − 1)	0	0	0	0	0	0
Government effectiveness	0	0	0	0	6	6
Rule of law	0	0	0	0	0	0
Control of corruption	0	0	0	0	0	0
Avg number of persons per household	0	2	0	0	0	2
Human Freedom (score)	0	2	0	2	4	8
Personal Freedom (score)	1	0	0	0	4	5
Economic Freedom (score)	0	0	0	0	6	6
**Religious Characteristics**						
Buddhist (% population)	2	0	2	0	4	8
Christian (% population)	1	4	2	4	2	13
Folk Religion (% population)	1	2	1	0	0	4
Jewish (% population)	0	0	0	0	0	0
Hindu (% population)	2	0	1	0	0	3
Irreligion (% population)	1	2	0	0	2	5
Muslim (% population)	0	2	0	2	2	6
Other Religion (% population)	0	0	1	0	2	3

**Signed Index of Importance of Socio-Economic Variables, Divided by Category**						
Variable	*Percentage of Models With Variable*					
	Y_1 (4)	$\tilde{Y}_1$ (8)	Y_2 (4)	$\tilde{Y}_2$ (8)	Y_0 (8)	*Total (32)*
**Healthcare Infrastructure**						
Physicians (/1000 people)	1	2	1	2	-2	4
Nurses and midwives (/1000 people)	0	-8	0	-4	0	-12
Hospital beds (/1000 people)	-1	-4	-3	-4	-4	-16
Essential health services (UHC) coverage index	2	0	2	0	4	8
**Health Statistics**						
Birth rate (crude, /1000 people)	-1	-2	0	-4	0	-7
Death rate (crude, /1000 people)	2	0	1	0	2	5
Life expectancy at birth (years)	0	0	1	0	0	1
Prevalence of diabetes between ages 20-79 (% population)	0	-4	-2	-6	-2	-14
Mortality from unsafe water, or sanitation, lack of hygiene combined (/100k people)	0	0	0	0	0	0
Completeness of death registration with cause-of-death information (%)	0	0	0	2	0	2
**Economic Health**						
GDP (per capita, PPP, $)	0	4	0	0	0	4
Unemployment rate (most recent available, % labor force)	0	0	0	0	0	0
Employment to population ratio for ages 15+ (modeled ILO estimate)	-2	-3	-2	-3	-4	-14
Domestic general government health expenditure (per capita, PPP, $)	1	2	3	2	4	12
Government lending/borrowing (% GDP)	0	0	1	-2	4	3
Income distribution (GINI index)	-2	-2	-2	-4	-4	-14
Trade (% GDP)	-1	2	-1	2	0	2
Number of airline passengers (per year)	4	0	3	0	0	7
Number of tourist arrivals (per year)	2	-2	2	0	4	6
**Demographic Structure**						
Population aged 65+ (% population)	1	2	1	4	0	8
Population aged $0-14$ (% population)	0*	0	0	0	0	0*
Population (total)	-2	0	-2	0	0	-4
Rural population (% population)	-1	4	-1	4	-2	4
International migrant stock (% population)	0	4	0	6	0	10
Population density (people per sq km)	0	4	1	4	0	9
**Environmental Health**						
Ecological footprint (gha/person)	1	0	1	0	2	4
Air pollution (avg P.M. 2.5 exposure per year)	0	0	0	0	0	0

*Population aged 0-14 was the first variable identified in LASSO for the geographically weighted $\tilde{Y}_1$, but it dropped out with the addition of the second variable and never returned. Further, it was not identified as important in any other models. Therefore we think it is unlikely that it is a significant variable.

Signed Index of Importance of Socio-Economic Variables, Divided by Category						
Variable	*Percentage of Models With Variable*					
	Y_1 (4)	$\tilde{Y}_1$ (8)	Y_2 (4)	$\tilde{Y}_2$ (8)	Y_0 (8)	*Total (32)*
**Societal Characteristics**						
Individuals using internet (% population)	0	-4	1	-2	-2	-7
Education level: Human capital index (0 – 1)	0	0	0	0	0	0
Government effectiveness	0	0	0	0	-6	-6
Rule of law	0	0	0	0	0	0
Control of corruption	0	0	0	0	0	0
Avg number of persons per household	0	2	0	0	0	2
Human Freedom (score)	0	-2	0	-2	4	0
Personal Freedom (score)	1	0	0	0	-4	-3
Economic Freedom (score)	0	0	0	0	-6	-6
**Religious Characteristics**						
Buddhist (% population)	-2	0	-2	0	-4	-8
Christian (% population)	1	4	2	4	2	13
Folk Religion (% population)	-1	2	-1	0	0	0
Jewish (% population)	0	0	0	0	0	0
Hindu (% population)	0	0	-1	0	0	-1
Irreligion (% population)	-1	-2	0	0	2	-1
Muslim (% population)	0	-2	0	-2	2	-2
Other Religion (% population)	0	0	-1	0	-2	-3

Table of Pooled Index of Importance of Socio-Economic Variables, Divided by Category				
Variable	*Number of Models With Variable*			
	Y_1 & $\tilde{Y}_1$ (12)	Y_2 & $\tilde{Y}_2$ (12)	Y_0 (8)	*Total (32)*
**Healthcare Infrastructure**				
Physicians (/1000 people)	3	3	2	8
Nurses and midwives (/1000 people)	8	4	0	12
Hospital beds (/1000 people)	5	7	4	16
Essential health services (UHC) coverage index	2	2	4	8
**Health Statistics**				
Birth rate (crude, /1000 people)	3	4	0	7
Death rate (crude, /1000 people)	2	1	2	5
Life expectancy at birth (years)	0	1	0	1
Prevalence of diabetes between ages 20-79 (% population)	4	8	2	14
Mortality from unsafe water, or sanitation, lack of hygiene combined (/100k people)	0	0	0	0
Completeness of death registration with cause-of-death information (%)	0	2	0	2
**Economic Health**				
GDP (per capita, PPP, $)	4	0	0	4
Unemployment rate (most recent available, % labor force)	0	0	0	0
Employment to population ratio for ages 15+ (modeled ILO estimate)	5	5	4	14
Domestic general government health expenditure (per capita, PPP, $)	3	5	4	12
Government lending/borrowing (% GDP)	0	3	4	7
Income distribution (GINI index)	4	6	4	14
Trade (% GDP)	3	3	0	6
Number of airline passengers (per year)	4	3	0	7
Number of tourist arrivals (per year)	8	10	4	22
**Demographic Structure**				
Population aged 65+ (% population)	3	5	0	8
Population aged $0-14$ (% population)	0*	0	0	0*
Population (total)	2	2	0	4
Rural population (% population)	5	5	2	12
International migrant stock (% population)	4	6	0	10
Population density (people per sq km)	4	5	0	9
**Environmental Health**				
Ecological footprint (gha/person)	1	1	2	4
Air pollution (avg P.M. 2.5 exposure per year)	0	0	4	4

*Population aged 0-14 was the first variable identified in LASSO for the geographically weighted $\tilde{Y}_1$, but it dropped out with the addition of the second variable and never returned. Further, it was not identified as important in any other models. Therefore we think it is unlikely that it is a significant variable.

Table of Pooled Index of Importance of Socio-Economic Variables, Divided by Category				
Variable	*Number of Models With Variable*			
	Y_1 & $\tilde{Y}_1$ (12)	Y_2 & $\tilde{Y}_2$ (12)	Y_0 (8)	*Total (32)*
**Societal Characteristics**				
Individuals using internet (% population)	4	3	2	9
Education level: Human capital index (0 – 1)	0	0	0	0
Government effectiveness	0	0	6	6
Rule of law	0	0	0	0
Control of corruption	0	0	0	0
Avg number of persons per household	2	0	0	2
Human Freedom (score)	2	2	4	8
Personal Freedom (score)	1	0	4	5
Economic Freedom (score)	0	0	6	6
**Religious Characteristics**				
Buddhist (% population)	2	2	4	8
Christian (% population)	5	6	2	13
Folk Religion (% population)	3	1	0	4
Jewish (% population)	0	0	0	0
Hindu (% population)	2	1	0	3
Irreligion (% population)	3	0	2	5
Muslim (% population)	2	2	2	6
Other Religion (% population)	0	1	2	3
